# Comparisons of High Intensity Interval Training and Continuous Training on Metabolomic Alteration and Cardiac Function in Male Adolescent Rats

**DOI:** 10.3389/fphys.2022.900661

**Published:** 2022-06-28

**Authors:** Molin Zheng, Chuanan Liu, Yuanyuan Lv, Jing Mi, Dan Qiu, Lingxiao He, Li Zhao

**Affiliations:** ^1^ Department of Exercise Physiology, Beijing Sport University, Beijing, China; ^2^ School of Competitive Sports, Beijing Sport University, Beijing, China; ^3^ Key Laboratory of Physical Fitness and Exercise, Ministry of Education, Beijing Sport University, Beijing, China; ^4^ School of Public Health, Xiamen University, Xiamen, China

**Keywords:** adolescent rats, cardiac function, continuous training, high intensity interval training, untargeted metabolomics

## Abstract

**Background:** Comparisons between high intensity interval training (HIIT) and continuous training (CT) regarding improvements of adolescents’ cardiac function are scarce and the preferred intensity for cardiac improvement with restricted myocardial damage remains unknown. This study conducted a 4-weeks training in male adolescent rats under moderate (MI) or high intensity (HI) HIIT and CT programs, aiming to discover and compare exercise-induced myocardial adaptations towards these two training methods.

**Methods:** 39 male adolescent Sprague-Dawley rats (aged 4 weeks) were randomly assigned to high intensity HIIT (HI-HIIT, *n* = 8), moderate intensity HIIT (MI-HIIT, *n* = 8), high intensity CT (HI-CT, *n* = 8), moderate intensity CT (MI-CT, *n* = 8) and sedentary control (SC, *n* = 7) groups. Rats in training groups were trained for 4 weeks and echocardiography was performed at baseline and after the final training. Serum creatine kinase myocardial band (CK-MB), cardiac troponin T (cTn-T) and untargeted metabolomics analysis were measured from blood samples collected 24 h after the final training.

**Results:** HIIT groups had greater cardiac output improvement than CT groups while no significant difference was found between the HI-HIIT and the MI-HIIT groups. HI-CT group showed higher serum CK-MB and cTn-T levels compared to MI-HIIT, MI-CT and control groups. Untargeted metabolomics analysis identified eleven HI-HIIT-related metabolites, five MI-HIIT-related metabolites and two HICT-related metabolites. The majority of the identified metabolites were phospholipid-related. Phosphatidylglyceride 18 level was significantly different between the HI-CT and MI-CT groups, and was negatively associated with cTn-T in CT groups.

**Conclusion:** HIIT and CT improve cardiac function of adolescent rats while the HIIT demonstrates better improvement and less myocardial damage. High and moderate training intensities in HIIT exert similar cardiac benefits. HI-CT induced myocardial damage might be associated with serum phospholipids.

## Introduction

Exercise has been well recognized as a beneficial approach in promoting heart function and combating cardiovascular diseases (Guasch and Nattel, 2013). Besides traditional continuous training (CT), high intensity interval training (HIIT) has been gaining popularity in recent years. The HIIT is a training form with combinations of short high-intensity bursts at the training phase and low-intensity exercises at the interval phase (Gibala et al., 2012; Tabata, 2019; Moniz et al., 2020). Therefore, the HIIT intensity is determined by multiple factors including intensity, duration and exercise patterns at both training and interval phases (Gibala et al., 2012). According to the Tabata protocol, an optimal duration ratio of high-intensity training to low-intensity interval phase should be 2:1 for its maximized energetical effect (Tabata, 2019).

Multiple studies have shown that the HIIT is beneficial for the cardiovascular system by increasing skeletal muscle oxidative capacity (assessed as maximal activity and content of cytochrome c oxidase) (Gibala et al., 2006), endothelial function (Tjønna et al., 2008) and maximum oxygen consumption (V̇O_2_max) (Guiraud et al., 2012). Moreover, when comparing with the CT, the HIIT demonstrates several advantages. For instance, the HIIT is more time-efficient than the CT in producing the same health benefits (Moniz et al., 2020). A recent study of obese young women showed that HIIT elicited similar improvement in cardiorespiratory fitness (evaluated as V̇O_2_max and blood lipids) as moderate intensity CT in only half the exercise time (Kong et al., 2016). A 6-weeks training on young adults also showed that HIIT induced similar V̇O_2_max, mitochondria function and lipid oxidation improvement as CT but with markedly less time commitment and lower training volume (Burgomaster et al., 2008). Previous research further demonstrated that HIIT outperformed CT in ameliorating brachial artery flow-mediated dilation (Ramos et al., 2015) and left ventricular morphology (Wisløff et al., 2007). Such advantages of HIIT over CT may be partly related to increased mitochondrial content (MacInnis et al., 2017).

Notably, current HIIT studies mainly focus on adults, comparisons of HIIT and CT effects on cardiovascular system in adolescents remain unknown. Moreover, although HIIT ameliorates cardiovascular disease-related biomarkers (e.g., blood pressure, glucose and total cholesterol) in adolescents (Eddolls et al., 2017), its impact on cardiac function and morphology have not been fully investigated. Considering the differences of cardiovascular responses between children and adults in both submaximal and maximal exercises (Turley, 1997), the cardiac adaptations to the HIIT and the CT might be different between adolescents and adults. Additionally, it remains to be answered whether the HIIT (usually over 80% of V̇O_2_max) (Moniz et al., 2020) is stringent for adolescents and will induce myocardial damage characterized by elevated serum creatine kinase myocardial band (CK-MB) and cardiac troponin (cTn) after excessive exercise dose (Weippert et al., 2016). An exploration on HIIT-induced cardiac changes and the comparisons with the CT can be helpful for exercise guidelines for adolescents.

Therefore, this research aimed to compare the training effects of HIIT and CT on cardiac function and morphology in adolescent rats. Following American College of Sports Medicine’s guidelines, moderate and high training intensities in each training form were applied (ACSM, 2017). To better understand exercise-induced metabolic changes, cardiac damage-related serum biomarkers were tested. Additionally, since the HIIT induces a faster fat oxidation shift than the CT with regards to metabolic adaptations (Horowitz and Klein, 2000; Talanian et al., 2007; Perry et al., 2008), untargeted metabolomics were performed to further explore metabolic differences behind the two training forms. We hypothesized that, due to the stringent intensity, the HIIT may induce more improvements on cardiac functions but with higher levels of cardiac damage-related biomarkers than the CT.

## Material and Methods

### Animals

39 male Sprague-Dawley rats (aged 4 weeks, Beijing HFK Bioscience Co., LTD., Beijing, China) were randomized into high training intensity HIIT group (HI-HIIT, *n* = 8), moderate training intensity HIIT group (MI-HIIT, *n* = 8), high training intensity CT group (HI-CT, *n* = 8), moderate training intensity CT group (MI-CT, *n* = 8) and sedentary control group (SC, *n* = 7). The rats were raised in a stable environmental condition (22 ± 2°C room temperature, 45% — 55% relative humidity, 12:12 h light-dark cycle) with *ad libitum* feeding. This research was approved by the ethics committee of the university (2015015) and followed the *Guiding Principles for Care and Use of Animals*.

### V̇O_2max_ Assessment

The oxygen consumption was measured by Oxymax Deluxe System (Columbus Instruments, Columbus, United States) and the V̇O_2max_ assessment followed the Bedford protocol (Bedford et al., 1979). Briefly, a rat was tested on a treadmill at an initial speed of 10 m/min with a gradient of 10°. The speed was gradually increased by 3 m/min every 3 min until the rat reached exhaustion or the oxygen consumption reached plateau. The final oxygen consumption by the end of the test was recorded as the V̇O_2max_ and the corresponding speed (vV̇O_2max_) was used as a surrogate to set exercise intensity. At the beginning of each week, two rats were randomly selected from exercise groups for the V̇O_2max_ assessment and the exercise intensity was adjusted according to the average value of vV̇O_2max_.

### Training Protocol

Before the formal training, rats in exercise groups (i.e., HI-HIIT, MI-HIIT, HI-CT, and MI-CT groups) received a 1-week (3 days/week) familiarization training which consisted of 5 min treadmill training at the speed of 7.5 m/min, 5 min at 10 m/min and 5 min at 12 m/min with a 1-min rest interval between each speed level.

The training protocols and intensities followed the guidelines of Buchheit and Laursen (Buchheit and Laursen, 2013), and exercise intervals followed Tabata’s protocol (Tabata, 2019). Specifically, the HI-HIIT group included 15 combined sets of one-min high intensity running exercise at 180% vV̇O_2max_ and two-min low intensity running exercise at 15% vV̇O_2max_. The MI-HIIT group also consisted of 15 combined sets and the exercise intensities were 140% and 35% vV̇O_2max_ for high and moderate intensity exercise, respectively. In the HI-CT group, rats were trained at 90% vV̇O_2max_ for 35 min. In the MI-CT group, rats were trained at 70% vV̇O_2max_ for 45 min. The rats in exercise groups were trained 3 days/week for 4 weeks and the SC group did not receive exercise training throughout the study. The design of the training protocol ensured that the rats in each exercise group completed the same training distance (i.e., 787.5 m in week 1, 859.95 m in week 2, 926.1 m in week 3 and 963.9 m in week 4). Detailed training protocols were presented in Supplementary Table S1.

### Echocardiographic Measurements

Cardiac function and morphology were evaluated by GE Vivid I ultrasound system (GE Healthcare, Atlanta, GA, United States) before and after the 4-weeks training with a 10 MHz linear array transducer. The measurement followed the method of Kutschka et al. (2007). Specifically, the rats were anesthetized with 2% isoflurane before the scan. Standard parasternal long and short axis images at the mid-ventricular level were obtained at a temporal resolution of approximately 200 frames per second. Heart rate, stroke volume, and left ventricular intraventricular diameter (LVID) (measured at diastolic and systolic phases) were measured. Cardiac output, ejection fraction and fractional shortening were also obtained by the ultrasound system.

### Serum Sample Collection and Cardiac Marker Measurement

To minimize the impact of an acute training session on circulating metabolites, abdominal aorta blood was collected 24 h after the completion of the final training. The collected blood was centrifuged, and serum samples were extracted and frozen at −80°C for blood test. Cardiac troponin T (cTn-T) and creatine kinase myocardial band (CK-MB) were measured by enzyme-linked immunoassay using Hitachi 7600 automatic biochemistry analyzer (Hitachi Ltd., Chiyoda-ku, Tokyo Japan).

### Untargeted Metabolomics Analysis

100 μL serum sample were transferred into a 1.5 ml centrifuge tube and mixed with 300 μL ice cold methanol. After a 30-min ultrasonic extraction, the sample was centrifuged at 12,000 rpm for 10 min. The supernatant was transferred into a new 1.5 ml centrifuge tube, concentrated in vacuo mixed with methanol for reconstitution. After a 10-min centrifugation, 10 μL supernatant was put into the liquid phase for metabolomics analysis. A quality control sample was created as a mixture of 10 μL supernatant from each of the tested samples.

Chromatographic separation was performed on a 1.8-µm HSS T3 column (Waters Corp, Milford, MA, United States; 150 × 3 mm) equipped with a UPLC system. The temperature of the column was 35°C and the flow rate was 0.3 ml/min. The mobile phase consisted of acetonitrile (Equate A) and 0.1% CH_3_COOH-H_2_O (Equate B). The elution gradient was 10 min 100% Equate B; 3 min 50% Equate A and 50% Equate B; 1 min 95% Equate A and 5% Equate B; and a return to 100% Equate B.

Mass spectrometry was performed by TripleTOF5600+ (AB SCIEX, Framingham, MA, United States) using an electrospray ionization (ESI) interface. Parameters of the analysis systems were: capillary voltage, 5000 V in ESI^+^ and 4500 V in ESI^−^; capillary temperature, 500°C; declustering potential, 60 V; collision energy, 35 V; collision energy spread, 15 V; mass spectrum scan range, 100–1,500 m/z; and scan mode, data independent acquisition (DIA) strategy.

The raw data were imported into MS-DIAL (version 2.76) (Tsugawa et al., 2015) for preprocessing, including peak extraction, noise removal, deconvolution and peak alignment. The preprocessed data were later annotated in three spectral databases: MassBank, Respect, and GNPS (14,951 records in total). The data were log-transformed (zero values were added by 0.001 before transformation) and standardized before between-group metabolite profile comparisons. *p* values were adjusted by Benjamini-Hochberg procedure.

### Statistics

Data were presented as mean ± standard deviation. Within-group comparison was performed by paired *t*-test to evaluate parameter changes pre- and post-training. Two-way repeated measures analysis of variance (ANOVA) was performed for between-group comparison with “groups” (i.e., HI-HIIT, MI-HIIT, HI-CT, MI-CT, and SC groups) as the between-group factor and “time” (i.e., pre- and post-training) as the repeated factor. One-way ANOVA was performed to analyze exercise associations with CK-MB and cTn-T. Tukey’s test was used as post-hoc test. Analyses were performed by R (version 4.1.1) (Team, 2021). A value of *p* < 0.05 was considered statistically significant.

## Results

### Cardiac Function and Morphology

**FIGURE 1:**
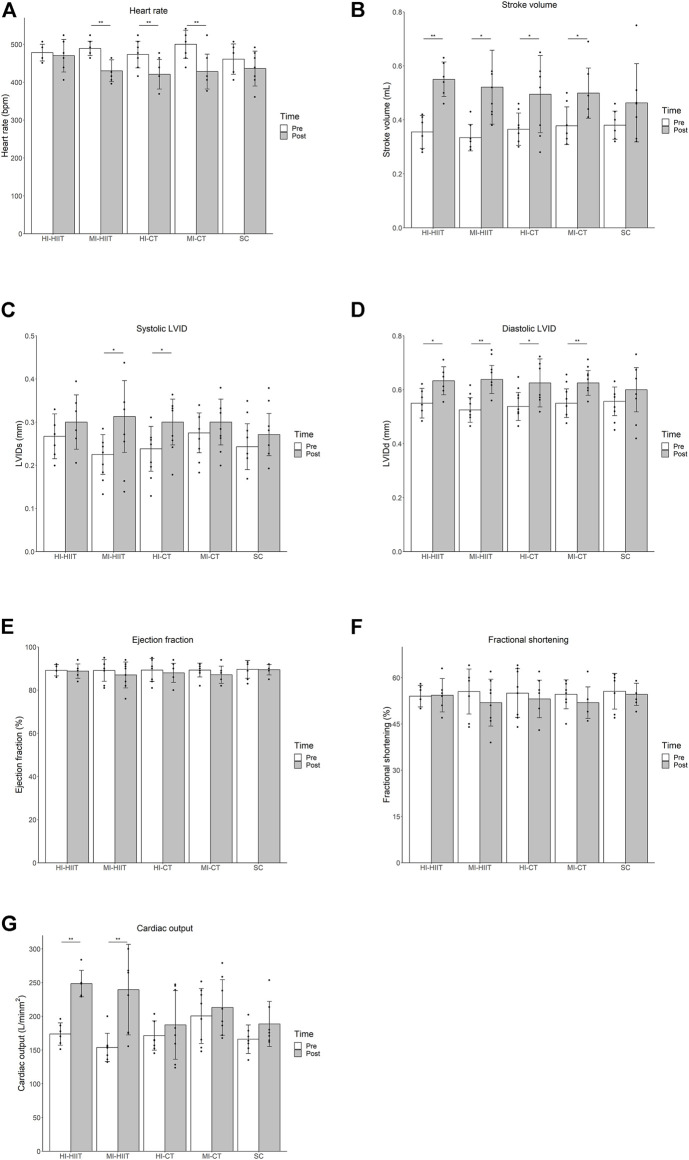
**(A)** Significant heart rate decline was found in the MI-HIIT, HI-CT and MI-CT group after training. **(B)** Stroke volume increased significantly in all exercise groups after training. **(C)** Systolic LVID increased in MI-HIIT and HI-CT groups after training. **(D)** Diastolic LVID increased in all exercise groups after training. **(E)** No significant change in ejection fraction was found in exercise groups after training. **(F)** No significant change in fractional shortening was found in exercise groups after training. **(G)** Significant increase in cardiac output was found in HIIT groups after training. HI-HIIT: high training intensity HIIT group; MI-HIIT: moderate training intensity HIIT group; HI-CT: high training intensity CT group; MI-CT: moderate training intensity CT group; SC: sedentary control group; LVID: left ventricular intraventricular diameter. *: *p* < 0.05; **p < 0.01.

Paired *t*-test within each exercise group showed that after the 4-weeks training, heart rate decreased in the MI-HIIT [(*p* < 0.001), HI-CT (*p* = 0.007) and MI-CT group (*p* = 0.004). Stroke volume and diastolic LVID increased in all exercise groups [HI-HIIT (*p* = 0.002, 0.042), MI-HIIT (*p* = 0.012, 0.007), HI-CT (*p* = 0.048, 0.041), MI-CT (*p* = 0.020, 0.003)]. Systolic LVID increased in the MI-HIIT (*p* = 0.021) and HI-CT (*p* = 0.011) group. HIIT groups had significant improvement in cardiac output [HI-HIIT (*p* < 0.001), MI-HIIT (*p* = 0.005)] while CT groups remained unchanged. No significant changes were found in ejection fraction and fractional shortening in all exercise groups (Table 1; Figure 1).

**TABLE 1:** Cardiac function and morphology in each group before and after training.

	HI-HIIT		MI-HIIT		HI-CT		MI-CT		SC	
	Pre	Post	Pre	Post	Pre	Post	Pre	Post	Pre	Post
Heart rate (bpm)	478 ± 22	470 ± 43	489 ± 19	430 ± 29**	473 ± 35	421 ± 39**	500 ± 37	428 ± 46**	461 ± 40	436 ± 46
Stroke volume (ml)	0.355 ± 0.061	0.550 ± 0.064**	0.334 ± 0.049	0.521 ± 0.137*	0.365 ± 0.060	0.495 ± 0.143*	0.378 ± 0.070	0.499 ± 0.093*	0.380 ± 0.052	0.463 ± 0.145
LVIDs (mm)	0.267 ± 0.052	0.300 ± 0.063	0.225 ± 0.046	0.313 ± 0.083*	0.238 ± 0.052	0.300 ± 0.053*	0.275 ± 0.046	0.300 ± 0.053	0.243 ± 0.053	0.271 ± 0.049
LVIDd (mm)	0.550 ± 0.055	0.633 ± 0.052*	0.525 ± 0.046	0.638 ± 0.052**	0.538 ± 0.052	0.625 ± 0.089*	0.550 ± 0.053	0.625 ± 0.046**	0.557 ± 0.053	0.600 ± 0.082
Ejection fraction (%)	89.2 ± 2.6	88.8 ± 3.3	89.1 ± 5.0	87.0 ± 6.0	89.3 ± 5.3	88.0 ± 4.4	89.3 ± 3.2	87.1 ± 4.0	89.6 ± 4.1	89.4 ± 2.4
Fractional shortening (%)	54.0 ± 3.5	54.3 ± 5.4	55.5 ± 7.3	51.9 ± 7.6	55.0 ± 7.9	53.1 ± 6.1	54.6 ± 4.7	51.9 ± 5.1	55.6 ± 5.8	54.6 ± 3.6
Cardiac output (L/min·m^2^)	173.7 ± 16.6	248.5 ± 19.6**	153.9 ± 21	239.5 ± 67.1**	171.5 ± 21.7	187.3 ± 50.9	200.5 ± 40.7	213.2 ± 41.3	166.1 ± 21.1	188.7 ± 33.4

* *p* < 0.05 compared to pre-training, **p < 0.01 compared to pre-training.

LVIDs, left ventricular intraventricular diameter at systolic phase; LVIDd, left ventricular intraventricular diameter at diastolic phase.

Repeated measures ANOVA among all exercise groups demonstrated decreased heart rate (F_(1,32)_ = 31.18, *p* < 0.001), and increased stroke volume (F_(1,32)_ = 43.39, *p* < 0.001), cardiac output (F_(1,32)_ = 24.67, *p* < 0.001) and left ventricular intraventricular diameters (diastolic phase F_(1,32)_ = 34.52, *p* < 0.001; systolic phase F_(1,32)_ = 15.42, *p* < 0.001) after training. Moreover, changes in heart rate, stroke volume and left ventricular intraventricular diameters did not show group-wise difference while cardiac output change differed among groups (F_(4,32)_ = 2.73, *p* = 0.046). Repeated measures ANOVA on each pair of groups showed similar improvement in cardiac output between the two HIIT groups (F_(1,12)_ = 0.18, *p* = 0.68) and the two CT groups (F_(1,14)_ = 0.01, *p* = 0.92). The HIIT groups demonstrated greater cardiac output improvement than the CT groups and the SC group while no significant difference in cardiac output change were found between the CT groups and the SC group.

### Myocardial Damage-Related Parameters

**FIGURE 2:**
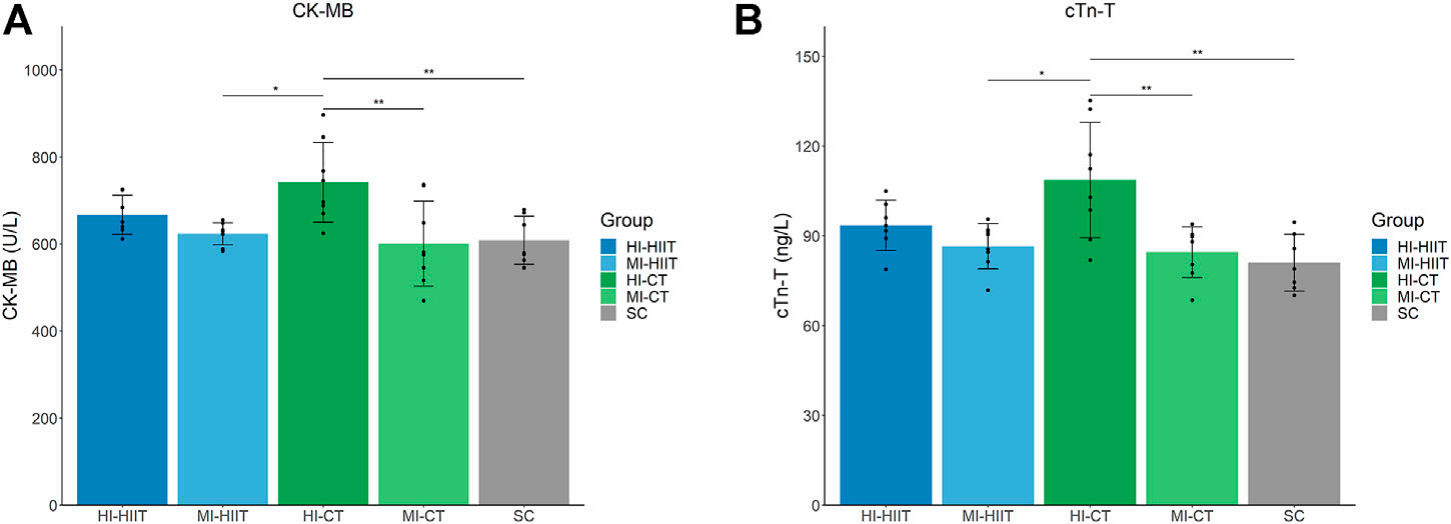
**(A)** Distribution of CK-MB in each group. HI-CT group demonstrated higher CK-MB than MI-HIIT (*p* = 0.015), MI-CT (*p* = 0.003) and SC (*p* = 0.007) groups. **(B)** Distribution of cTn-T in each group. HI-CT group demonstrated higher cTn-T than MI-HIIT (*p* = 0.005), MI-CT (*p* = 0.002) and SC (*p* = 0.001) groups. HI-HIIT: high training intensity HIIT group; MI-HIIT: moderate training intensity HIIT group; HI-CT: high training intensity CT group; MI-CT: moderate training intensity CT group; SC: sedentary control group; cTn-T: Cardiac troponin T; CK-MB: creatine kinase myocardial band.

Significant differences were found in CK-MB (F_(4,33)_ = 5.46, *p* = 0.002) and cTn-T (F_(4,33)_ = 6.91, *p* < 0.001). The HI-CT group demonstrated the highest CK-MB and cTn-T levels than other groups. Tukey’s post-hoc test showed significant higher CK-MB and cTnT values in HI-CT compared to MI-HIIT (*p* = 0.015 and 0.005, respectively), MI-CT (*p* = 0.003 and 0.002, respectively) and SC (*p* = 0.007 and 0.001, respectively) groups (Table 2; Figure 2).

**TABLE 2:** CK-MB and cTn-T in each group.

	HI-HIIT	MI-HIIT	HI-CT	MI-CT	SC
CK-MB (U/L)*	682.0 ± 60.3	623.3 ± 25.4	741.7 ± 91.8	600.9 ± 97.6	608.2 ± 55.1
cTn-T (ng/L)*	92.34 ± 8.46	86.53 ± 7.52	108.64 ± 19.27	84.56 ± 8.43	81.00 ± 9.40

* Significant difference in group factor analyzed by one-way ANOVA.

### Untargeted Metabolomics

The raw data obtained by mass spectrometry were preprocessed by MS-DIAL and PCA was applied for quality control (Supplemental Figure S1). Peak values of the extracted data were compared with 14,951 records in three databases (MassBank, Respect, GNPS) and 371 metabolites were annotated.

**FIGURE 3:**
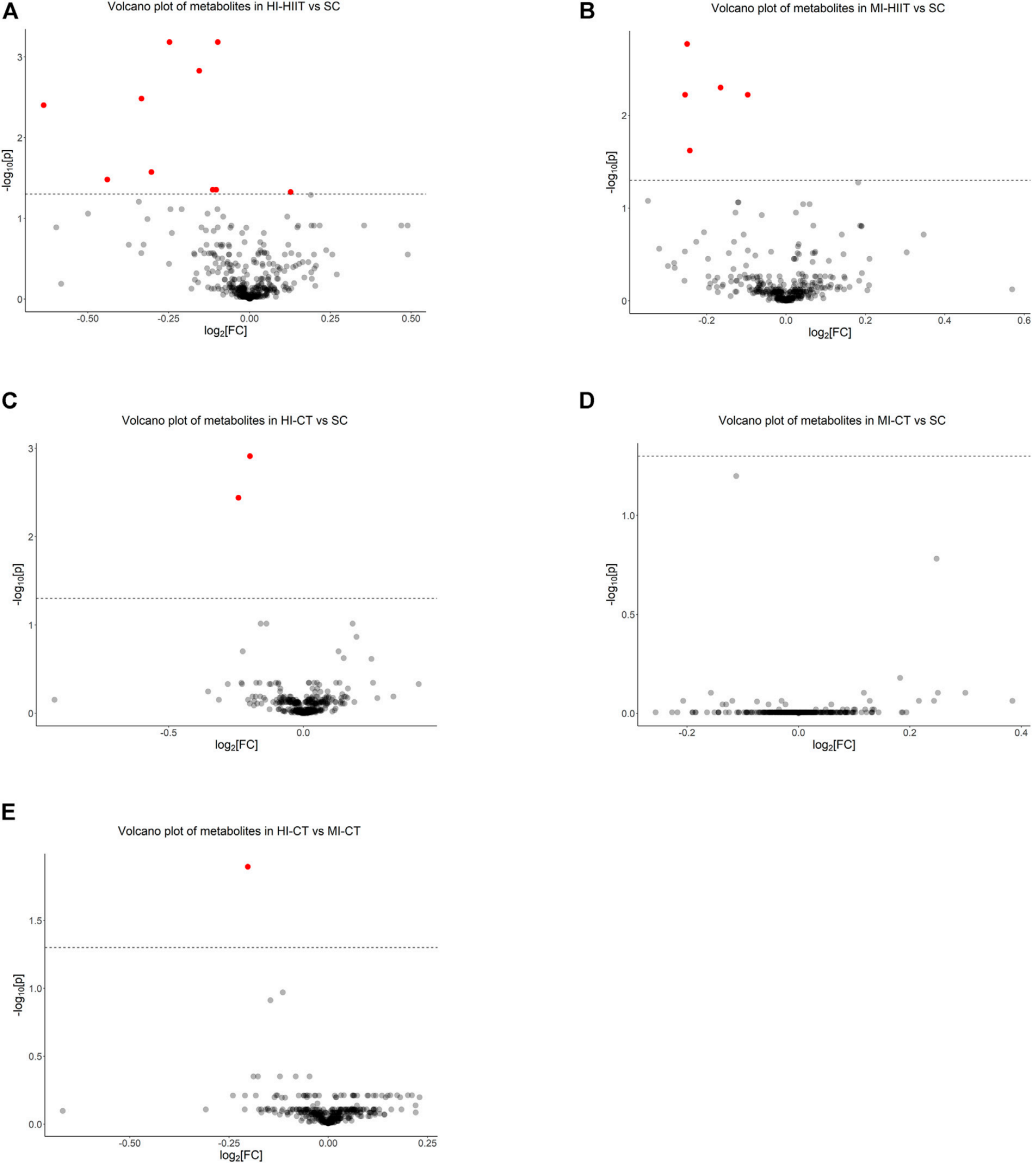
Metabolomics analysis identified **(A)** 11 significant metabolites between HI-HIIT and SC, **(B)** five significant metabolites between MI-HIIT and SC, **(C)** two significant metabolites between HI-CT and SC, **(D)** no significant metabolite between MI-CT and SC, and **(E)** one significant metabolite between HI-CT and MI-CT groups. The dash line represents the cutoff value at a negative log-transformed significance level of 0.05 (i.e., $-\log_{10}\left[0.05\right]=1.3$ ). HI-HIIT: high training intensity HIIT group; MI-HIIT: moderate training intensity HIIT group; HI-CT: high training intensity CT group; MI-CT: moderate training intensity CT group; SC: sedentary control group.

Metabolite comparisons identified 11 metabolites that demonstrated significantly different levels between the HI-HIIT and SC groups (Figure 3A, Supplementary Material Table S2) and 10 metabolites demonstrated lower levels in the HI-HIIT group than the SC group. Among the identified metabolites, five metabolites (two phosphatidylinositols, two phosphatidylcholines and one phosphatidylglyceride) were lipid-related.

Five metabolites were identified between the MI-HIIT and SC groups. Four metabolites (two phosphatidylinositols, one phosphoinositide and one phosphatidylglyceride) were lipid-related and one metabolite (2′-Deoxyadenosine-5′-monophosphate) was related to DNA synthesis (Figure 3B; Supplementary Table S3).

Two metabolites (one phosphatidylinositol and one phosphatidylglyceride) were identified in comparisons between the HI-CT and SC groups (Figure 3C; Supplementary Table S4). No significant differences in metabolite levels were found between the MI-CT and SC groups. Notable, three metabolites (two phosphatidylinositols and 2′-Deoxyadenosine-5′-monophosphate) were found in both HIIT groups but not in CT groups. One phosphatidylglyceride (phosphatidylglyceride 18) was found in all comparisons. No significant differences of metabolite levels were found between the MI-CT and SC groups (Figure 3D).

Since the HI-CT group demonstrated significantly higher CK-MB and cTn-T levels than the MI-CT group, extra metabolite comparisons were performed between CT groups and phosphatidylglyceride 18 level was found to be significantly lower in the HI-CT group (Figure 3E; Supplementary Table S5). Further association analysis showed that phosphatidylglyceride 18 was negatively associated with cTn-T in CT groups (*p* = 0.016) but not in HIIT groups (*p* = 0.687) while no significant association was found between phosphatidylglyceride 18 and CK-MB.

Two-way ANOVA with exercise groups (i.e., HIIT and CT) and training intensity (i.e., HI and MI) as two factors identified five metabolites (two phosphatidylinositols, AMP, uric acid and gamma-Glutamylglutamine) that were different between the two exercise groups (*p* value adjusted) (Supplementary Table S6). Specifically, higher levels of phosphatidylinositols, AMP and gamma-Glutamylglutamine, and a lower level of uric acid were associated with the HIIT compared to the CT. Four metabolites (two phosphatidylglycerides, one phosphatidylcholine and one phosphatidylcholine) in lipid sub-pathways were significantly downregulated in the high training intensity compared to the moderate one (*p* value adjusted) (Supplementary Table S6).

## Discussion

To the best of our knowledge, this is the first study to compare the HIIT and CT effects on cardiac function and morphology in adolescent rats. We found decreased heart rate, and increased stroke volume and left ventricular intraventricular diameters in exercise groups after a 4-weeks training. HIIT groups demonstrated greater cardiac output improvement than CT groups. Moreover, high training intensity CT group induced higher levels of serum myocardial damage-related markers (i.e., CK-MB and cTn-T) compared to moderate intensity HIIT and CT groups. Untargeted metabolomics analysis further identified one phosphatidylglyceride (phosphatidylglyceride 18) that was negatively associated with cTn-T in CT groups.

### Exercise Improves Cardiac Function and Morphology

Physical exercise has been well recognized as a convenient and economic way of improving cardiovascular conditions. In the current study, the heart rate in exercise groups decreased after training, which was a sign of improved health condition since higher resting heart rate has been independently associated with increased risks of all-cause mortality (Jensen et al., 2013; Zhang et al., 2016). Moreover, exercise-induced improvements in stroke volume and left ventricular intraventricular diameters provided supporting evidence for the beneficial effects of exercise on cardiovascular system.

Notably, although changes in ejection fraction, heart rate and stroke volume were not significantly different among exercise groups, adaptations in cardiac output were significantly larger in the HIIT groups than the CT ones. These findings were partially in line with the study of Verboven et al. (Verboven et al., 2019), who found similar improvements in ejection fraction and cardiac volumes between rats that received a 13-weeks of HIIT or CT. However, Verboven et al. failed to find significant differences in cardiac output improvements between training groups. A possible explanation might be the difference in HIIT intensity as Verboven et al. used a speed of 18 m/min and an inclination of 30° while we used a speed of over 35 m/min and an inclination of 10°. Yet, the increased cardiac capillarization density in the HIIT group identified by Verboven et al. might also help to explain the increased cardiac output in our study (Verboven et al., 2019). Moreover, MacInnis et al. reported that the HIIT induced greater increases in mitochondrial content (measured as elevated maximal citrate synthase activity and mitochondrial respiration) than the CT with the same exercise volume (MacInnis et al., 2017). Although MacInnis’ findings were based on skeletal muscle tissue, considering the holistic effect of exercise, increased mitochondrial content might also happen in myocardium. Besides possible increase in capillarization density and mitochondrial content, the HIIT might also enhance cardiac function by modifying circulating phosphatidylcholine levels which were inversely associated with vascular condition and risk of cardiovascular diseases (Stegemann et al., 2014; Paapstel et al., 2018). Our study was in line with previous findings and further showed that the HIIT was superior than the CT in improving cardiac function among adolescent rats while the insignificant difference in stroke volume change might be due to the short intervention period.

### Associations Between Exercise and Myocardial Damage

To evaluate myocardial damage after the intervention, serum CK-MB and cTn-T were measured in the study. CK-MB and cTn-T are two biomarkers commonly used to assist diagnoses of myocardial infarction (Task Force et al., 2007) while cTn-T demonstrates higher sensitivity in detecting myocardial injury than CK-MB (Sobki et al., 2000). CK-MB is one of the three creatine kinase isoenzymes, and is primarily found in heart muscle cells. It has been used as a biomarker for myocardial injury detection in the past decades (Wu et al., 2020) before cTn-T is suggested. cTn-T is a contractile protein of cardiac muscle. It may release to peripheral blood after heart damage, and it is also a biomarker of myocardial oxidative stress (König et al., 2007). Elevated CK-MB and cTn-T levels can be found after prolonged or strenuous endurance exercise, and possible mechanisms might include increased membrane permeability and myocardial cell necrosis (Shave et al., 2010). Surprisingly, our results reveal opposite phenomenon to our hypothesis, CK-MB and cTn-T levels were similar in HIIT groups and moderate intensity CT group while higher levels of these two myocardial injury-related markers were found in high intensity CT group in comparison with other exercise groups. Considering that circulating cTn-T usually returns to the resting level within 24 h after exercise (Gresslien and Agewall, 2016; Baker et al., 2019), our findings indicated that the HI-CT was a less favorable exercise form in cardiovascular exercise for adolescents due to the prolonged myocardial damage. In order to understand possible mechanisms behind the finding of less myocardial damage in the HIIT, we introduced untargeted metabolomics to explore potentially relevant biomarkers.

Previous metabolomics studies showed that acute exercise was associated with high oxidative stress and increased phosphatidylcholine levels that might impair utilization of cardiac fatty acids and lead to inflammation-related metabolic disorders (Paapstel et al., 2018; Wu et al., 2022). Yet, our results showed that long-term exercise could exert a favorable effect on cardiovascular system by reducing phosphatidylcholine concentrations. Analyses on metabolite levels between the HI-CT and MI-CT groups identified a downregulated phosphatidylglyceride (phosphatidylglyceride 18) in the HI-CT group. Phosphatidylglyceride is a fundamental component of intracellular membrane, particularly mitochondrial and microsomal membranes (Morita and Terada, 2015). Previous studies have reported that exercise training may induce phosphatidylglyceride degradation and reduce lipid accumulation, preventing the occurrence of cardiomyopathy and heart failure (Borg et al., 2012). Lower plasma phosphatidylglyceride levels were also found in mice after swimming endurance training compared to the control group (Tham et al., 2018). Our finding of declined serum phosphatidylglyceride in all exercise groups was consistent with previous studies. Notably, we found a significantly lower phosphatidylglyceride 18 level in the HI-CT compared to the MI-CT. Combining with our finding that the HI-CT group demonstrated a considerably high level of cTn-T, the lower phosphatidylglyceride might be an indicator of a greater extent of myocardial damage compared to other exercise groups. Such assumption is supported by the negative association between phosphatidylglyceride 18 and cTn-T in CT groups rather than HIIT groups. The underlying mechanism between phosphatidylglyceride 18 and cardiac injury is currently not understood but is worthy of further study since changes in circulating phospholipids might play a role in lipid remodeling of the heart, energy metabolism and myocardial function.

### HIIT-Related Metabolomics

To identify potential molecular and pathway alterations associated with the HIIT, we compared serum metabolites in HIIT groups with that in the control group and identified four phospholipid-related metabolites including phosphatidylglycerides and phosphatidylinositols. Phospholipids are involved in multiple important metabolic pathways such as cellular signaling, intracellular trafficking and insulin resistance (Jiang, 2012). Previous lipidomics research has identified linoleic acid-containing phosphatidylcholine, sphingomyelin and docosahexanoic acid-containing phosphatidylcholine as chronic exercise-induced lipids (Goto-Inoue et al., 2013). Multiple lipid metabolites (e.g., BHBA and decanoylcarnitine) were also identified in resistance and endurance training (Morville et al., 2020). The identification of HIIT-related metabolites implied a potential connection between HIIT and lipid pathways. Further studies are needed to explore such association.

Notably, among the five identified metabolites that were associated with exercise types (Supplementary Table S6), decreased AMP and increased uric acid were significantly associated with the HIIT compared to the CT. Since decreased ATP content activates the AMP degradation pathway with uric acid as the end product (Francis and Hamrick, 1984), the HIIT-related associations in AMP and uric acid implied a higher energy consumption in the HIIT compared to the CT. Among the rest three identified metabolites, two were involved in fatty acid metabolism sub-pathway and one was from the gamma-glutamyl amino acid sub-pathway. Despite the difference in exercise forms, the lower levels of these three metabolites in the HIIT compared to the CT were similar to the findings of high-level endurance athletes demonstrating lower levels of metabolites involved in fatty acid metabolism and gamma-glutamyl amino acid pathways than moderate-level peers (Al-Khelaifi et al., 2018), indicating that the HIIT might be associated with a higher fitness level. Yet, further research are needed to elucidate the potential functions of these three metabolites in HIIT.

### HIIT Outperforms CT in Improving Cardiovascular Function of Adolescent Rats

Since the HIIT intensity is at a relatively high level by the CT standard, we hypothesized that the HIIT might induce greater myocardial damage than the CT. However, our results showed that the HIIT induced more cardiac output and demonstrated less myocardial impairment (as indicated by CK-MB and cTn-T) than the CT under the same relative training volume (i.e., 31.5 min·vV̇O_2max_). Therefore, the HIIT might be better than the CT when assigning cardiovascular exercise to adolescent. Additionally, when the HIIT is applied, both high and moderate intensities are fine for training since they exert similar impact on cardiac improvement. Yet, when the CT is used, a moderate intensity is better than a high intensity since the latter induces more cardiac damage.

### Limitations

Although we found greater improvement in cardiac function induced by the HIIT in comparison with the CT, we did not explore underlying mechanisms behind such finding. Moreover, the study was performed on male rat, additional research is needed to verify our findings in female rats and human. Despite that our rats were raised under the same condition, future studies with nutrition and food intake tracking might be helpful to draw a more convincing conclusion.

In conclusion, our research suggests that the HIIT might outperform the CT in improving cardiovascular condition of adolescent rats as the HIIT induced a greater cardiac output adaptation after a 4-weeks training and less myocardial damages 24 h after exercise. The cardiovascular benefits of moderate and high intensity HIIT were similar, but high intensity CT induced more myocardial damage than moderate intensity CT. Such myocardial damage in the CT might be associated with phospholipid metabolism.

## Data Availability

The original contributions presented in the study are included in the article/Supplementary Material, further inquiries can be directed to the corresponding authors.

## References

[B1] ACSM (2017). ACSM's Complete Guide to Fitness & Health, 2E. Champaign: Human Kinetics.

[B2] Al-Khelaifi F. Diboun I. Donati F. Botrè F. Alsayrafi M. Georgakopoulos C. (2018). A Pilot Study Comparing the Metabolic Profiles of Elite-Level Athletes from Different Sporting Disciplines. Sports Med. - Open 4 (1), 2. 10.1186/s40798-017-0114-z 29305667 PMC5756230

[B3] Baker P. Leckie T. Harrington D. Richardson A. (2019). Exercise-induced Cardiac Troponin Elevation: An Update on the Evidence, Mechanism and Implications. IJC Heart & Vasc. 22, 181–186. 10.1016/j.ijcha.2019.03.001 PMC6437282 30963092

[B4] Bedford T. G. Tipton C. M. Wilson N. C. Oppliger R. A. Gisolfi C. V. (1979). Maximum Oxygen Consumption of Rats and its Changes with Various Experimental Procedures. J. Appl. Physiology 47 (6), 1278–1283. 10.1152/jappl.1979.47.6.1278 536299

[B5] Borg M. L. Omran S. F. Weir J. Meikle P. J. Watt M. J. (2012). Consumption of a High-Fat Diet, but Not Regular Endurance Exercise Training, Regulates Hypothalamic Lipid Accumulation in Mice. J. Physiology 590 (17), 4377–4389. 10.1113/jphysiol.2012.233288 PMC3473292 22674717

[B6] Buchheit M. Laursen P. B. (2013). High-Intensity Interval Training, Solutions to the Programming Puzzle. Sports Med. 43 (5), 313–338. 10.1007/s40279-013-0029-x 23539308

[B7] Burgomaster K. A. Howarth K. R. Phillips S. M. Rakobowchuk M. MacDonald M. J. McGee S. L. (2008). Similar Metabolic Adaptations during Exercise after Low Volume Sprint Interval and Traditional Endurance Training in Humans. J. Physiology 586 (1), 151–160. 10.1113/jphysiol.2007.142109 PMC2375551 17991697

[B8] Eddolls W. T. B. McNarry M. A. Stratton G. Winn C. O. N. Mackintosh K. A. (2017). High-Intensity Interval Training Interventions in Children and Adolescents: A Systematic Review. Sports Med. 47 (11), 2363–2374. 10.1007/s40279-017-0753-8 28643209 PMC5633633

[B9] Francis K. T. Hamrick M. E. (1984). Exercise and Uric Acid: Implication in Cardiovascular Disease. J. Orthop. Sports Phys. Ther. 6 (1), 34–38. 10.2519/jospt.1984.6.1.34 18806378

[B10] Gibala M. J. Little J. P. Van Essen M. Wilkin G. P. Burgomaster K. A. Safdar A. (2006). Short-term Sprint Intervalversustraditional Endurance Training: Similar Initial Adaptations in Human Skeletal Muscle and Exercise Performance. J. Physiology 575 (3), 901–911. 10.1113/jphysiol.2006.112094 PMC1995688 16825308

[B11] Gibala M. J. Little J. P. MacDonald M. J. Hawley J. A. (2012). Physiological Adaptations to Low-Volume, High-Intensity Interval Training in Health and Disease. J. Physiology 590 (5), 1077–1084. 10.1113/jphysiol.2011.224725 PMC3381816 22289907

[B12] Goto-Inoue N. Yamada K. Inagaki A. Furuichi Y. Ogino S. Manabe Y. (2013). Lipidomics Analysis Revealed the Phospholipid Compositional Changes in Muscle by Chronic Exercise and High-Fat Diet. Sci. Rep. 3 (1), 3267. 10.1038/srep03267 24253370 PMC3834553

[B13] Gresslien T. Agewall S. (2016). Troponin and Exercise. Int. J. Cardiol. 221, 609–621. 10.1016/j.ijcard.2016.06.243 27420587

[B14] Guasch E. Nattel S. (2013). CrossTalk Proposal: Prolonged Intense Exercise Training Does Lead to Myocardial Damage. J. Physiology 591 (20), 4939–4941. 10.1113/jphysiol.2013.257238 PMC3810793 24130313

[B15] Guiraud T. Nigam A. Gremeaux V. Meyer P. Juneau M. Bosquet L. (2012). High-Intensity Interval Training in Cardiac Rehabilitation. Sports Med. 42 (7), 587–605. 10.2165/11631910-000000000-00000 22694349

[B16] Horowitz J. F. Klein S. (2000). Lipid Metabolism during Endurance Exercise. Am. J. Clin. Nutr. 72 (2 Suppl. l), 558S–63S. 10.1093/ajcn/72.2.558S 10919960

[B17] Jensen M. T. Suadicani P. Hein H. O. Gyntelberg F. (2013). Elevated Resting Heart Rate, Physical Fitness and All-Cause Mortality: a 16-year Follow-Up in the Copenhagen Male Study. Heart 99 (12), 882–887. 10.1136/heartjnl-2012-303375 23595657 PMC3664385

[B18] Jiang Y. (2012). IGF-1 Mediates Exercise-Induced Phospholipid Alteration in the Murine Skin Tissues. J. Nutr. Food Sci. S2 (01), 1–6. 10.4172/2155-9600.S2-003

[B19] Kong Z. Fan X. Sun S. Song L. Shi Q. Nie J. (2016). Comparison of High-Intensity Interval Training and Moderate-To-Vigorous Continuous Training for Cardiometabolic Health and Exercise Enjoyment in Obese Young Women: A Randomized Controlled Trial. PLOS ONE 11 (7), e0158589. 10.1371/journal.pone.0158589 27368057 PMC4930190

[B20] König D. Neubauer O. Nics L. Kern N. Berg A. Bisse E. (2007). Biomarkers of Exercise-Induced Myocardial Stress in Relation to Inflammatory and Oxidative Stress. Exerc Immunol. Rev. 13, 15–36. 18198658

[B21] Kutschka I. Sheikh A. Y. Sista R. Hendry S. L. Chun H. J. Hoyt G. (2007). A Novel Platform Device for Rodent Echocardiography. ILAR J. 49 (2), E1–E7. 10.1093/ilar.49.2.e1 18506056

[B22] MacInnis M. J. Zacharewicz E. Martin B. J. Haikalis M. E. Skelly L. E. Tarnopolsky M. A. (2017). Superior Mitochondrial Adaptations in Human Skeletal Muscle after Interval Compared to Continuous Single-Leg Cycling Matched for Total Work. J. Physiol. 595 (9), 2955–2968. 10.1113/JP272570 27396440 PMC5407978

[B23] Moniz S. C. Islam H. Hazell T. J. (2020). Mechanistic and Methodological Perspectives on the Impact of Intense Interval Training on Post‐exercise Metabolism. Scand. J. Med. Sci. Sports 30 (4), 638–651. 10.1111/sms.13610 31830334

[B24] Morita S.-y. Terada T. (2015). Enzymatic Measurement of Phosphatidylglycerol and Cardiolipin in Cultured Cells and Mitochondria. Sci. Rep. 5 (1), 11737. 10.1038/srep11737 26122953 PMC4485230

[B25] Morville T. Sahl R. E. Moritz T. Helge J. W. Clemmensen C. (2020). Plasma Metabolome Profiling of Resistance Exercise and Endurance Exercise in Humans. Cell. Rep. 33 (13), 108554. 10.1016/j.celrep.2020.108554 33378671

[B26] Paapstel K. Kals J. Eha J. Tootsi K. Ottas A. Piir A. (2018). Inverse Relations of Serum Phosphatidylcholines and Lysophosphatidylcholines with Vascular Damage and Heart Rate in Patients with Atherosclerosis. Nutr. Metabolism Cardiovasc. Dis. 28 (1), 44–52. 10.1016/j.numecd.2017.07.011 28986077

[B27] Perry C. G. R. Heigenhauser G. J. F. Bonen A. Spriet L. L. (2008). High-intensity Aerobic Interval Training Increases Fat and Carbohydrate Metabolic Capacities in Human Skeletal Muscle. Appl. Physiol. Nutr. Metab. 33 (6), 1112–1123. 10.1139/h08-097 19088769

[B28] Ramos J. S. Dalleck L. C. Tjonna A. E. Beetham K. S. Coombes J. S. (2015). The Impact of High-Intensity Interval Training versus Moderate-Intensity Continuous Training on Vascular Function: a Systematic Review and Meta-Analysis. Sports Med. 45 (5), 679–692. 10.1007/s40279-015-0321-z 25771785

[B29] Shave R. Baggish A. George K. Wood M. Scharhag J. Whyte G. (2010). Exercise-Induced Cardiac Troponin Elevation. J. Am. Coll. Cardiol. 56 (3), 169–176. 10.1016/j.jacc.2010.03.037 20620736

[B30] Sobki S. H. Saadeddin S. M. Habbab M. A. (2000). Cardiac Markers Used in the Detection of Myocardial Injury. Saudi Med. J. 21 (9), 843–846. 11376361

[B31] Stegemann C. Pechlaner R. Willeit P. Langley S. R. Mangino M. Mayr U. (2014). Lipidomics Profiling and Risk of Cardiovascular Disease in the Prospective Population-Based Bruneck Study. Circulation 129 (18), 1821–1831. 10.1161/circulationaha.113.002500 24622385

[B32] Tabata I. (2019). Tabata Training: One of the Most Energetically Effective High-Intensity Intermittent Training Methods. J. Physiol. Sci. 69 (4), 559–572. 10.1007/s12576-019-00676-7 31004287 PMC10717222

[B33] Talanian J. L. Galloway S. D. R. Heigenhauser G. J. F. Bonen A. Spriet L. L. (2007). Two Weeks of High-Intensity Aerobic Interval Training Increases the Capacity for Fat Oxidation during Exercise in Women. J. Appl. physiology 102 (4), 1439–1447. 10.1152/japplphysiol.01098.2006 17170203

[B34] Task Force M. Thygesen K. Alpert J. S. White H. D. White H. D. Jaffe A. S. (2007). Universal Definition of Myocardial Infarction. Circulation 116 (20), 2634–2653. 10.1093/eurheartj/ehm35510.1161/CIRCULATIONAHA.107.187397 17951284

[B35] Team R. C. (2021). R: A Language and Environment for Statistical Computing. Vienna, Austria: R Foundation for Statistical Computing.

[B36] Tham Y. K. Bernardo B. C. Huynh K. Ooi J. Y. Y. Gao X. M. Kiriazis H. (2018). Lipidomic Profiles of the Heart and Circulation in Response to Exercise versus Cardiac Pathology: A Resource of Potential Biomarkers and Drug Targets. Cell. Rep. 24 (10), 2757–2772. 10.1016/j.celrep.2018.08.017 30184508

[B37] Tjønna A. E. Lee S. J. Rognmo Ø. Stølen T. O. Bye A. Haram P. M. (2008). Aerobic Interval Training versus Continuous Moderate Exercise as a Treatment for the Metabolic Syndrome. Circulation 118 (4), 346–354. 10.1161/CIRCULATIONAHA.108.772822 18606913 PMC2777731

[B38] Tsugawa H. Cajka T. Kind T. Ma Y. Higgins B. Ikeda K. (2015). MS-DIAL: Data-independent MS/MS Deconvolution for Comprehensive Metabolome Analysis. Nat. Methods 12 (6), 523–526. 10.1038/nmeth.3393 25938372 PMC4449330

[B39] Turley K. R. (1997). Cardiovascular Responses to Exercise in Children. Sports Med. 24 (4), 241–257. 10.2165/00007256-199724040-00003 9339493

[B40] Verboven M. Cuypers A. Deluyker D. Lambrichts I. Eijnde B. O. Hansen D. (2019). High Intensity Training Improves Cardiac Function in Healthy Rats. Sci. Rep. 9 (1), 5612. 10.1038/s41598-019-42023-1 30948751 PMC6449502

[B41] Weippert M. Divchev D. Schmidt P. Gettel H. Neugebauer A. Behrens K. (2016). Cardiac Troponin T and Echocardiographic Dimensions after Repeated Sprint vs. Moderate Intensity Continuous Exercise in Healthy Young Males. Sci. Rep. 6 (1), 24614. 10.1038/srep24614 27090032 PMC4835763

[B42] Wisløff U. Støylen A. Loennechen J. P. Bruvold M. Rognmo Ø. Haram P. M. (2007). Superior Cardiovascular Effect of Aerobic Interval Training versus Moderate Continuous Training in Heart Failure Patients. Circulation 115 (24), 3086–3094. 10.1161/CIRCULATIONAHA.106.675041 17548726

[B43] Wu Y.-W. Ho S. K. Tseng W.-K. Yeh H.-I. Leu H.-B. Yin W.-H. (2020). Potential Impacts of High-Sensitivity Creatine Kinase-MB on Long-Term Clinical Outcomes in Patients with Stable Coronary Heart Disease. Sci. Rep. 10 (1), 5638. 10.1038/s41598-020-61894-3 32221337 PMC7101408

[B44] Wu L. Wang J. Cao X. Tian Y. Li J. (2022). Effect of Acute High-Intensity Exercise on Myocardium Metabolic Profiles in Rat and Human Study via Metabolomics Approach. Sci. Rep. 12 (1), 1–12. 10.1038/s41598-022-10976-5 35473956 PMC9042871

[B45] Zhang D. Shen X. Qi X. (2016). Resting Heart Rate and All-Cause and Cardiovascular Mortality in the General Population: a Meta-Analysis. Can. Med. Assoc. J. 188 (3), E53–E63. 10.1503/cmaj.150535 26598376 PMC4754196

