# Supplementary figures and images for: Comparisons of High Intensity Interval Training and Continuous Training on Metabolomic Alteration and Cardiac Function in Male Adolescent Rats
Source: Front Physiol. 2022 Jun 28;13:900661. doi: 10.3389/fphys.2022.900661 (PMC9274303; doi:10.3389/fphys.2022.900661)

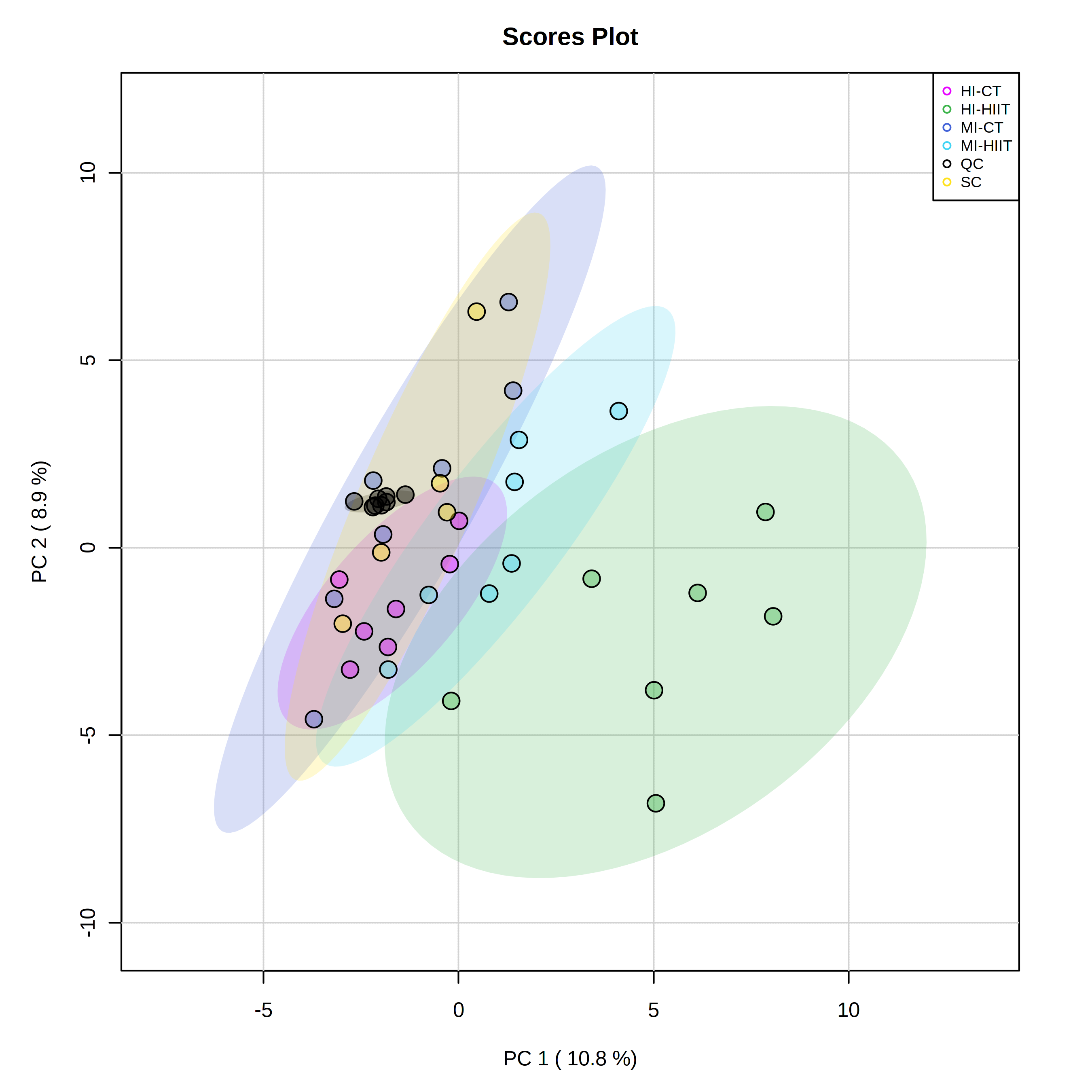

Supplement: Supplementary file 1 [file Image1.TIFF]
