# Supplementary material for: Comparisons of High Intensity Interval Training and Continuous Training on Metabolomic Alteration and Cardiac Function in Male Adolescent Rats
Source: Front Physiol. 2022 Jun 28;13:900661. doi: 10.3389/fphys.2022.900661 (PMC9274303; doi:10.3389/fphys.2022.900661)
Supplement: Supplementary file 2 [file Table1.DOCX]

**Supplementary material**

Table 1 Training protocols

|  | HI-HIIT | MI-HIIT | HI-CT | MI-CT |  |
| --- | --- | --- | --- | --- | --- |
| Familiarization week | 7.5 m/min for 5min, rest for 1min; 10 m/min for 5min, rest for 1 min; 12 m/min for 5 min. | | | |  |
| Week 1 | Warm-up: 10 m/min, gradient 10° for 10 min.  1 trial: gradient 10°, 45 m/min for 1 min, 3.75 m/min for 2 min.  Repeated for 15 trials. | Warm-up: 10 m/min, gradient 10° for 10 min.  1 trial: gradient 10°, 35 m/min for 1 min, 8.75 m/min for 2 min.  Repeated for 15 trials. | Warm-up: 10 m/min, gradient 10° for 10 min. Training: 22.5 m/min, gradient 10°, for 35 min. | Warm-up: 10 m/min, gradient 10° for 10 min. Training: gradient 10°, 17.5 m/min for 45 min. |  |
|  |  |  |  |  |  |
|  |  |  |  |  |  |
|  |  |  |  |  |  |
|  |  |  |  |  |  |
| Week 2 | Warm-up: 10 m/min, gradient 10° for 10 min.  1 trial: gradient 10°, 49.14 m/min for 1 min, 4.095 m/min for 2 min.  Repeated for 15 trials. | Warm-up: 10 m/min, gradient 10° for 10 min.  1 trial: gradient 10°, 38.22 m/min for 1 min, 9.555 m/min for 2 min.  Repeated for 15 trials. | Warm-up: 10 m/min, gradient 10° for 10 min. Training: gradient 10°, 24.57 m/min for 35 min. | Warm-up: 10 m/min, gradient 10° for 10 min. Training: gradient 10°, 19.11 m/min for 45 min. |  |
|  |  |  |  |  |  |
|  |  |  |  |  |  |
|  |  |  |  |  |  |
|  |  |  |  |  |  |
| Week 3 | Warm-up: 10 m/min, gradient 10° for 10 min.  1 trial: gradient 10°, 52.92 m/min for 1 min, 4.41 m/min for 2 min.  Repeated for 15 trials. | Warm-up: 10 m/min, gradient 10° for 10 min.  1 trial: gradient 10°, 41.16 m/min for 1 min, 10.29 m/min for 2 min.  Repeated for 15 trials. | Warm-up: 10 m/min, gradient 10° for 10 min. Training: gradient 10°, 24.57 m/min for 35 min. | Warm-up: 10 m/min, gradient 10° for 10 min. Training: gradient 10°, 20.58 m/min for 45 min. |  |
|  |  |  |  |  |  |
|  |  |  |  |  |  |
|  |  |  |  |  |  |
|  |  |  |  |  |  |
| Week 4 | Warm-up: 10 m/min, gradient 10° for 10 min.  1 trial: gradient 10°, 55.08 m/min for 1 min, 4.59 m/min for 2 min.  Repeated for 15 trials. | Warm-up: 10 m/min, gradient 10° for 10 min.  1 trial: gradient 10°, 42.84 m/min for 1 min, 10.71 m/min for 2 min.  Repeated for 15 trials. | Warm-up: 10 m/min, gradient 10° for 10 min. Training: gradient 10°, 27.54 m/min for 35 min. | Warm-up: 10 m/min, gradient 10° for 10 min. Training: gradient 10°, 21.42 m/min for 45 min. |  |
|  |  |  |  |  |  |
|  |  |  |  |  |  |
|  |  |  |  |  |  |
|  |  |  |  |  |  |

Table 2 Significant metabolites between HI-HIIT and SC groups

| Metabolite ID | Metabolite name | Ontology | p.value | p.adjusted (FDR) | FC | Log2(FC) |
| --- | --- | --- | --- | --- | --- | --- |
| meta368 | Phosphatidylinositol 16 | Phosphatidylinositols | 1.84E-06 | 0.00065196 | 0.84249 | -0.24727 |
| meta347 | Phosphatidylglyceride 18 | Phosphatidylglycerols | 3.51E-06 | 0.00065196 | 0.93419 | -0.09821 |
| meta367 | Phosphatidylinositol 17 | Phosphatidylinositols | 1.20E-05 | 0.00148 | 0.89772 | -0.15566 |
| meta171 | GalNAcFucGlcNAcGA-I | Oligosaccharides | 3.53E-05 | 0.0032697 | 0.79324 | -0.33417 |
| meta344 | Phosphatidylcholine 15 | Phosphatidylcholines | 5.33E-05 | 0.003957 | 0.6432 | -0.63665 |
| meta364 | PC (16:0/11,12-EpETE) |  | 0.000432 | 0.026681 | 0.81034 | -0.30341 |
| meta134 | 2'-Deoxyadenosine-5'-monophosphate | Purine 2'-deoxyribonucleoside monophosphates | 0.000621 | 0.032919 | 0.73729 | -0.43969 |
| meta288 | FA 18:1+3O | Oxidized fatty acids | 0.001106 | 0.044118 | 0.9241 | -0.11388 |
| meta261 | Xanthosine | Purine nucleosides | 0.00115 | 0.044118 | 0.93084 | -0.10339 |
| meta262 | Xanthosine | Purine nucleosides | 0.001189 | 0.044118 | 0.93095 | -0.10322 |
| meta102 | gamma-Glutamyl-cysteine | N-acyl-alpha amino acids | 0.001395 | 0.047062 | 1.092 | 0.12692 |

Table 3 Significant metabolites between MI-HIIT and SC groups

| Metabolite ID | Metabolite name | Ontology | p.value | p.adjusted (FDR) | FC | Log2(FC) |
| --- | --- | --- | --- | --- | --- | --- |
| meta367 | Phosphatidylinositol 17 | Phosphatidylinositols | 4.56E-06 | 0.00169 | 0.84125 | -0.2494 |
| meta371 | PI (18:0/12-HETE) |  | 2.7E-05 | 0.005001 | 0.8918 | -0.16521 |
| meta368 | Phosphatidylinositol 16 | Phosphatidylinositols | 6.11E-05 | 0.005966 | 0.83823 | -0.25458 |
| meta347 | Phosphatidylglyceride 18 | Phosphatidylglycerols | 6.43E-05 | 0.005966 | 0.93505 | -0.09688 |
| meta134 | 2'-Deoxyadenosine-5'-monophosphate | Purine 2'-deoxyribonucleoside monophosphates | 0.000322 | 0.023926 | 0.84524 | -0.24256 |

Table 4 Significant metabolites between HI-CT and SC groups

| Metabolite ID | Metabolite name | Ontology | p.value | p.adjusted (FDR) | FC | Log2(FC) |
| --- | --- | --- | --- | --- | --- | --- |
| meta371 | PI(18:0/12-HETE) |  | 3.3E-06 | 0.001223 | 0.87123 | -0.19888 |
| meta347 | Phosphatidylglyceride 18 | Phosphatidylglycerols | 1.95E-05 | 0.003624 | 0.84588 | -0.24147 |

Table 5 Significant metabolites between HI-CT and MI-CT groups

| Metabolite ID | Metabolite name | Ontology | p.value | p.adjusted (FDR) | FC | Log2(FC) |
| --- | --- | --- | --- | --- | --- | --- |
| meta347 | Phosphatidylglyceride 18 | Phosphatidylglycerols | 3.43E-05 | 0.01271 | 0.8689 | -0.20273 |

Table 6 Significant metabolites between HIIT and CT groups in two-way ANOVA

| Metabolite ID | Metabolite name | Ontology | Exercise (HIIT) estimate | Exercise (HIIT) p.adjusted | Intensity (HI) estimate | Intensity (HI) p.adjusted | Exercise (HIIT) × Intensity (HI) estimate | Exercise (HIIT) × Intensity (HI) p.adjusted |
| --- | --- | --- | --- | --- | --- | --- | --- | --- |
| meta367 | Phosphatidylinositol 17 | Phosphatidylinositols | -1.451 | < 0.001 | -0.732 | 0.019 | 1.323 | 0.595 |
| meta347 | Phosphatidylglyceride 18 | Phosphatidylglycerols | -1.304 | < 0.001 | -1.226 | 0.001 | 1.267 | 0.710 |
| meta295 | AMP | Purine ribonucleoside monophosphates | -1.193 | 0.007 | -0.764 | 0.242 | 0.785 | 0.710 |
| meta60 | Uric acid | Xanthines | 0.424 | 0.012 | 0.036 | 0.960 | -0.372 | 0.553 |
| meta118 | gamma-Glutamylglutamine | Dipeptides | -0.678 | 0.027 | -0.224 | 0.771 | 0.850 | 0.423 |

Two-way ANOVA with the CT group as the reference exercise group and the moderate intensity as the reference intensity. HIIT: high intensity interval training group; HI: high intensity

Supplemental figure 1 legend

Figure 1 PCA scores plot

Quality control assessment is performed by PCA. In the PCA score plot, QC samples (coloured in pink) are grouped in the middle (with a small variation), indicating that the testing instrument is stable during the mass spectrometry sequence. Therefore, subsequent data analysis can be performed. Notably, there are overlapping in confidence interval (marked as ellipse areas), indicating that the current two principal components cannot clearly separate the five groups.
